# The *Staphylococcus aureus* Extracellular Adherence Protein Eap Is a DNA Binding Protein Capable of Blocking Neutrophil Extracellular Trap Formation

**DOI:** 10.3389/fcimb.2018.00235

**Published:** 2018-07-09

**Authors:** Janina Eisenbeis, Mona Saffarzadeh, Henrik Peisker, Philipp Jung, Nicolas Thewes, Klaus T. Preissner, Mathias Herrmann, Virginie Molle, Brian V. Geisbrecht, Karin Jacobs, Markus Bischoff

**Affiliations:** ^1^Institute of Medical Microbiology and Hygiene, Saarland University, Homburg, Germany; ^2^Department of Biochemistry, Medical Faculty, Justus-Liebig-University, Giessen, Germany; ^3^Experimental Physics, Saarland University, Saarbrücken, Germany; ^4^Laboratoire de Dynamique des Interactions Membranaires Normales et Pathologiques, Centre National de la Recherche Scientifique, UMR 5235, Université de Montpellier, Montpellier, France; ^5^Department of Biochemistry and Molecular Biophysics, Kansas State University, Manhattan, KS, United States

**Keywords:** *Staphylococcus aureus*, extracellular adherence protein, DNA aggregation, atomic force microscopy, neutrophil extracellular traps, innate immunity

## Abstract

The extracellular adherence protein (Eap) of *Staphylococcus aureus* is a secreted protein known to exert a number of adhesive and immunomodulatory properties. Here we describe the intrinsic DNA binding activity of this multifunctional secretory factor. By using atomic force microscopy, we provide evidence that Eap can bind and aggregate DNA. While the origin of the DNA substrate (e.g., eukaryotic, bacterial, phage, and artificial DNA) seems to not be of major importance, the DNA structure (e.g., linear or circular) plays a critical role with respect to the ability of Eap to bind and condense DNA. Further functional assays corroborated the nature of Eap as a DNA binding protein, since Eap suppressed the formation of “neutrophil extracellular traps” (NETs), composed of DNA-histone scaffolds, which are thought to function as a neutrophil-mediated extracellular trapping mechanism. The DNA binding and aggregation activity of Eap may thereby protect *S. aureus* against a specific anti-microbial defense reaction from the host.

## Introduction

*Staphylococcus aureus* is an opportunistic pathogen and a major cause of acute, as well as, chronic skin and soft tissue infections (SSTIs) (Moet et al., 2007). A major threat of this bacterium is its ability to adhere and form large multicellular structures called biofilms on various implanted medical devices (O'Gara, 2007). Biofilm formation by *S. aureus* is a complex process that commonly includes the formation of an extracellular polymeric matrix including polysaccharide intercellular adhesin (PIA), protein to protein interactions, and the incorporation of extracellular DNA (eDNA); which are all used by multiplying *S. aureus* cells to form and to remain within the multilayered structure (O'Gara, 2007; Payne and Boles, 2016). β-hemolysin has been identified as a factor that can bind to eDNA (Huseby et al., 2010), however, this exotoxin is not produced by most clinical *S. aureus* isolates due to the insertion of a prophage into the β-hemolysin encoding *hlb* gene (Goerke et al., 2009).

Another *S. aureus* factor which may serve as binding partner for eDNA on the bacterial surface is extracellular adherence protein (Eap), a member of the “secretable expanded repertoire adhesive molecules” (SERAM) protein family (Chavakis et al., 2005). This basic protein is expressed by *S. aureus* in a growth phase-dependent manner (Joost et al., 2009), and secreted in large quantities into the extracellular milieu (Eisenbeis et al., 2017), from which it partially rebinds to the bacterial surface through the surface-bound neutral phosphatase (Nptase) and other yet unidentified cell wall molecules (Palma et al., 1999; Flock and Flock, 2001).

Secreted Eap has been shown to interact with a number of host cell matrix components, plasma proteins, and cellular receptors with a binding preference for extracellular cell matrix (ECM) super-structures (Hansen et al., 2006). Eap demonstrated function as a potent anti-inflammatory and anti-angiogenic factor (Chavakis et al., 2007). This multifunctional protein is also involved in biofilm formation under iron-limiting conditions (Johnson et al., 2008) or in the presence of serum (Thompson et al., 2010), however, its molecular function in biofilm formation of *S. aureus* remains largely unknown. Depending on the *S. aureus* strain background, this cationic protein consists of four to six similar, but not identical, repeats of about 100 amino acid residues each; these are designated EAP modules, which are connected by short 9–12 residues long linker regions (Hammel et al., 2007).

Binding of Eap to multiple soluble and extracellular matrix host ligands is thought to support the adhesion of *S. aureus* to host tissue, particularly in the context of inflammation and wounding (Hansen et al., 2006; Bur et al., 2013). Moreover, our preliminary experiments indicated that Eap might also interact with extracellular nucleic acids (RNA and DNA), thereby providing another targeted binding of *S. aureus* to sites of host cell injury and stress (Dumont, 1959; Gould et al., 2015). An important biological setting in which *S. aureus* may encounter large quantities of eDNA are neutrophil extracellular traps (NETs). Upon stimulation by invading pathogens and/or other stimuli, activated neutrophils may enter a cell-death program, in which nuclear, granular, and plasma membranes dissolve in an ordered manner, thereby releasing decondensed chromatin, decorated with granular proteins, into the extracellular space in order to entrap the invading microorganisms (Papayannopoulos and Zychlinsky, 2009; Azzouz et al., 2018). *S. aureus* may counteract this process by several means: the pathogen secretes Eap to block the activities of neutrophil serine proteases (Stapels et al., 2014), which are of major importance for NET formation (Stapels et al., 2015), and it also provides extracellular nucleases to degrade NETs (Berends et al., 2010).

Given, (i) the elongated structure of Eap with several surface-exposed binding sites, capable of participating in the interactions with diverse ligands (Hammel et al., 2007), (ii) its cationic charge in neutral solutions, (iii) its adhesive properties, and (iv) the fact that Eap is highly expressed in serum grown *S. aureus* biofilms (Thompson et al., 2010), we hypothesized that Eap might contribute to binding of bacteria to eDNA. By applying atomic force microscopy (AFM), we demonstrate here for the first time that Eap specifically binds to linearized DNA, aggregates this type of nucleic acid, and thereby is capable of interfering with NETosis by a novel mechanism.

## Materials and methods

### Bacterial strains and Eap preparations

The bacterial strains used in this study are listed in Table 1. *S. aureus* strains were routinely grown on Trypticase Soy Agar II with 5% Sheep Blood (Becton Dickinson, Heidelberg, Germany) at 37°C. *E. coli* strains were grown on Luria Bertani agar (Becton Dickinson), supplemented with 25 μg/ml kanamycin when needed. Eap was collected and purified from *S. aureus* strain Newman and its *nuc1 nuc2* derivative M0746N1 (Kaito et al., 2011) as described in Athanasopoulos et al. (2006) with the following modifications: *S. aureus* strains were grown in Modified B-Broth (Ohlsen et al., 1997) for 20 h at 37°C and 150 rpm with a culture to flask volume of 1:4. Cell suspensions were centrifuged at 5.525 × g and 4°C for 15 min, and cell pellets were washed with phosphate buffered saline (PBS pH 7.2; Thermo Fisher, Dreieich, Germany). Cell wall associated proteins were subsequently obtained by lithium chloride extraction, and the extracts adsorbed onto SP Sepharose (Amersham-Pharmacia, Freiburg, Germany). After stepwise elution with increasing NaCl concentrations, pooled eluted fractions were sterile filtered and further purified by cation exchange chromatography on a Mono S 5/50 GL column (Sigma-Aldrich, Munich, German) followed by gel filtration using a Superdex 75 HR 10/30 column (Sigma-Aldrich) operated on a BioLogic DuoFlow chromatography system (Bio-Rad, Munich, Germany). Protein concentrations and purity of the products were checked by SDS-PAGE and Bradford assay, respectively. Purified Eap samples were found to be free of detectable endotoxin. Eap and the Eap homologs EapH1 and EapH2 of Mu50 were additionally produced recombinant as His-tagged proteins in *Escherichia coli* and purified by using nickel affinity chromatography as described in Geisbrecht et al. (2005), Xie et al. (2006).

**Table 1:** Strains used in this study.

**Strain**	**Relevant genotype and phenotype*^a^***	**Reference or source**
***S. aureus***		
Newman	Laboratory strain (ATCC 25904), high level Eap producer	Duthie, 1952
M0746N1	Newman *nuc1::aph, nuc2::*pT1160; Cm^R^, Km^R^	Kaito et al., 2011
***E. coli***		
BL21(DE3)	F^−^*ompT hsdS*_B_(${\text{r}}_{\text{B}}^{-}$ ${\text{m}}_{\text{B}}^{-}$) *gal dcm* (DE3)	Novagen
BL834(DE3)	F^−^*ompT hsdS*_B_(${\text{r}}_{\text{B}}^{-}$ ${\text{m}}_{\text{B}}^{-}$) *gal dcm met* (DE3)	Novagen

a *Abbreviations are as follows: Cm^R^, chloramphenicol resistant; Km^R^, kanamycin resistant*.

### DNA sources

Herring sperm DNA (10164142), phage lambda DNA (D3779), and pBR322 (N3033L) DNA were purchased from Invitrogen (Karlsruhe, Germany), Sigma-Aldrich, and New England Biolabs (Frankfurt, Germany), respectively. To test the influences of a circular or linear state of DNA, and the nature of the DNA end on degradation by Eap, pBR322 was digested with the restriction enzymes *Bam*HI, *Eco*RV, and *Pst*I (Thermo Fisher), respectively, following the instructions of the manufacturer. The 1.4-kb DNA fragment was amplified by PCR using primer pair MBH492 (5′-CCTGAACAACCTGATGAGCC-3′)/MBH493 (5′-ACCCTATTTTTTCGCCAAGCC-3′), and chromosomal DNA from *S. aureus* strain Newman (Duthie, 1952) as template.

### Electrophoretic mobility shift assay

Fifty Nanogram of DNA (a 1.4-kb DNA fragment amplified by PCR using primer pair MBH492/MBH493 and chromosomal DNA from *S. aureus* strain Newman) was co-incubated with increasing concentrations of Eap (0, 1, 5, 10, 20, and 40 μg/ml; 0, 16, 80, 160, 320, and 640 nM) in PBS for 5 min at 37°C. Subsequently, DNA/Eap solutions were mixed 5:1 (v/v) with loading buffer [10 mM Tris-HCl [pH 7.6], 0.15% orange G, 60% glycerol] supplemented with 1 μl of a 1:100 dilution of SYBR® Gold nucleic acid gel stain (S11494; Invitrogen), and immediately subjected to agarose gel electrophoresis as described in Sambrook and David (2001). Gel images were recorded with the Gel Doc™ XR+ gel documentation system (Bio-Rad, Munich, Germany), and band intensities determined using the ImageLab software package 4.0.1 (Bio-Rad). The GeneRuler™ 1 kb DNA ladder (SM0311; Thermo Fisher) served as DNA size marker.

### DNase activity assays

To analyze the DNA degrading capacity of Eap, a number of DNase assays were performed. At first, the DNase-Activity ELISA ORG 590 (ORGENTEC Diagnostika, Mainz, Germany) was used according to the manufacturer's recommendations. In brief, 3 μg/ml of Eap were diluted 1:10 with sample buffer prior to the assay. To each well, 100 μl of calibrators, controls and pre-diluted Eap were added and incubated for 1 h at 37°C. After several washings, 100 μl of the enzyme conjugate solution including a DNase substrate antibody labeled with horseradish peroxidase (HRP) was added and incubated for 15 min at room temperature. Wells were washed again three times with wash solution, before the HRP substrate 3,3′,5,5′- tetramethylbenzidine (TMB) was added. After 15 min of incubation, the reaction was stopped and the optical density was read at 450 nm. The result was calculated using a bi-chromatic measurement with a reference at 600–690 nm. PCR grade water served as negative control, and 12.5 mKunitz units (mKuU) of DNase I served as positive control.

In a second assay, 50 ng of DNA (a 1.4-kb DNA fragment amplified by PCR using primer pair MBH492/MBH493 and chromosomal DNA from *S. aureus* strain Newman) was co-incubated with different concentrations of Eap (10, 20, and 40 μg/ml) in RPMI 1640 (Thermo Fisher) for 30 min at 37°C. Additionally, 50 ng of DNA was mixed with DNase I (0.4 mKuU) and co-incubated for 5, 15, and 30 min at 37°C, respectively. Subsequently, DNA/Eap and DNA/DNase I solutions were mixed with one volume of phenol/chloroform/isoamyl alcohol (25:24:1; Thermo Fisher), vortexed and centrifuged for 5 min at 16,000 × g. The DNA containing upper phases were carefully removed and mixed 5:1 (v/v) with loading buffer, and immediately subjected to agarose gel electrophoresis. Gels were subsequently incubated for 15 min in a 1x SYBR® Gold nucleic acid gel stain solution as described by the manufacturer, and gel images were recorded with the Gel DocTM XR+ gel documentation system.

Finally, the fluorescence based DNase detection kit (Jena Biosciences, Jena, Germany) was used according to the manufacturer's recommendations. Briefly, a master mix containing a dual labeled fluorescent DNase probe (a 27 bp oligonucleotide labeled at its 5′-end with fluorescein and its 3′-end with the quencher BHQ-1®), reaction buffer, and ROX® reference dye was mixed with Eap (10 and 40 μg/ml) and different amounts of a DNase I standard (2 and 0.2 mKuU), respectively. Solutions were placed into a real time fluorescence reader (Abi 7000; ThermoFisher) and incubated for 30 min at 37°C. Fluorescence signals at 520 and 601 nm were recorded after every minute, and FAM-signals were evaluated in relation to the ROX reference signals.

### Preparation of the substrate for DNA combing

Silicon (Si) wafers were used as substrates since they feature a very low roughness (0.09 nm) and are easily available in consistently good quality with a known surface chemistry. Si wafers (Siltronic AG, Burghausen, Germany) have a native silicon oxide layer (*d* = 1.7 nm). The wafers were rendered hydrophobic by self-assembly of a CH_3_ terminated monolayer of octadecyltrichlorosilane (OTS) molecules following a standard protocol (Lessel et al., 2015). Hydrophobized Si wafers displayed a surface roughness of 0.12 nm and an advancing (receding) water contact angle of 111° (107°).

### DNA combing

Molecular combing of DNA was performed according to the following procedure: A chemically modified CH3 terminated silicon wafer was immersed into a PBS/DNA solution (100 ng/ml DNA dissolved in PBS) with 0.5 μg/ml Eap added followed by a 5 min incubation time at room temperature. DNA fibers bind to the chemically modified, hydrophobic surface by one or both of their ends in a pH-dependent manner (Allemand et al., 1997). The wafer was then pulled out perpendicular with a slow and constant speed (*v* = 300 μm/s) using a xyz-axis motor-drive manipulator (MM-92B, Narashige Group, Tokyo, Japan). The receding meniscus stretches the anchored DNA molecules onto the hydrophobic surface as it applies a constant force on them. This rapid process results in irreversibly fixed DNA fibers and has the major advantage that DNA fibers are aligned in parallel on the surface. The stretching factor is constant (2 kb ~ 800 nm).

### AFM measurements

Atomic force microscopy (AFM) was carried out in air on a Bioscope Catalyst (Bruker, France), operating in ScanAsyst® mode using ScanAsyst-Air cantilevers (nominal spring constant 0.5 N/m, Bruker, France) and a tip velocity <5 μm/s. The images were processed and DNA length measured using the open source Gwyddion Software (Version 2.36). SigmaPlot11 (Systat Software GmbH, Erkrath, Germany) was used for length histogram generation.

### Human neutrophil isolation

Isolation of human neutrophils was conducted according to a previously published protocol (Saffarzadeh et al., 2012). Briefly, venous blood was collected from healthy donors in EDTA tubes and layered onto a double gradient that was formed by layering an equal volume of histopaque-1077 over histopaque-1119 (Sigma-Aldrich). Following centrifugation at 700 × g for 30 min, the granulocyte fraction was concentrated at the 1077/1119 interphase. These cells were aspirated and washed once with PBS. The remaining erythrocytes in that fraction were subsequently lysed by hypotonic shock using distilled water, and neutrophils were resuspended in phenol red-free RPMI 1640 (Invitrogen).

### Immunofluorescence microscopy

1 × 10^5^ neutrophils were seeded onto coverslips in 8-chamber slides (Nunc, Thermo Scientific, Germany) and treated with 25 nM phorbol 12-myristate 13-acetate (PMA, Sigma-Aldrich), lipopolysaccharides (LPS) from *Escherichia coli* 0111:B4 (Sigma-Aldrich), and spermine–nitric oxide complex (NO) (Sigma Aldrich), respectively, to induce NET-formation. Subsequently, cells were challenged with increasing concentrations of Eap (0, 1, 2.5, and 10 μg/ml) or DNase I (10 U/ml; Thermo Fisher), and incubated for 3 h at 37°C in presence of 5% CO_2_. In a second approach, neutrophils were pre-incubated for 2 h with PMA prior to the addition of Eap for 1 h. To visualize NETs, samples were fixed with 1% paraformaldehyde, blocked with 3% bovine serum albumin (BSA) and incubated with primary mouse anti-DNA/histone H1 antibody (Millipore, Germany) followed by detection with secondary antibody coupled to Alexa Fluor 555 donkey anti-mouse IgG (Invitrogen). The DNA/histone antibody has a very high affinity for decondensated chromatin in NETs in comparison to 4′,6-diamidino-2-phenylindole (DAPI) (Saffarzadeh et al., 2012, 2014). DAPI (Invitrogen) was used for nuclear DNA detection. Slides were mounted with ProLong Gold antifade reagent (Invitrogen). Cell images were taken with a fluorescence microscope (Leica Microsystems, Wetzlar, Germany), and NETs were quantified based on the area of DNA/histone antibody derived signals per 100 cells detected by DAPI, using the imaging software Fiji (Schindelin et al., 2012).

### Evaluation of apoptosis and necrosis

In order to investigate whether Eap may induce cell death in neutrophils, the PromoKine Apoptotic/Necrotic/Healthy Cells Detection Kit (PromoCell, Heidelberg, Germany) was used. Neutrophils were incubated for 3 h with Eap (10 or 20 μg/ml), and the percentage of apoptosis and necrosis was evaluated using FITC-Annexin, Ethidium homodimer III, and Hoechst 33342 according to the manufacturer's instructions.

### Statistics

Statistical significance was assessed using the GraphPad Prism software package 6.01. *p* values <0.05 were considered significant.

## Results and discussion

### Eap binds to DNA

**Figure 1:**
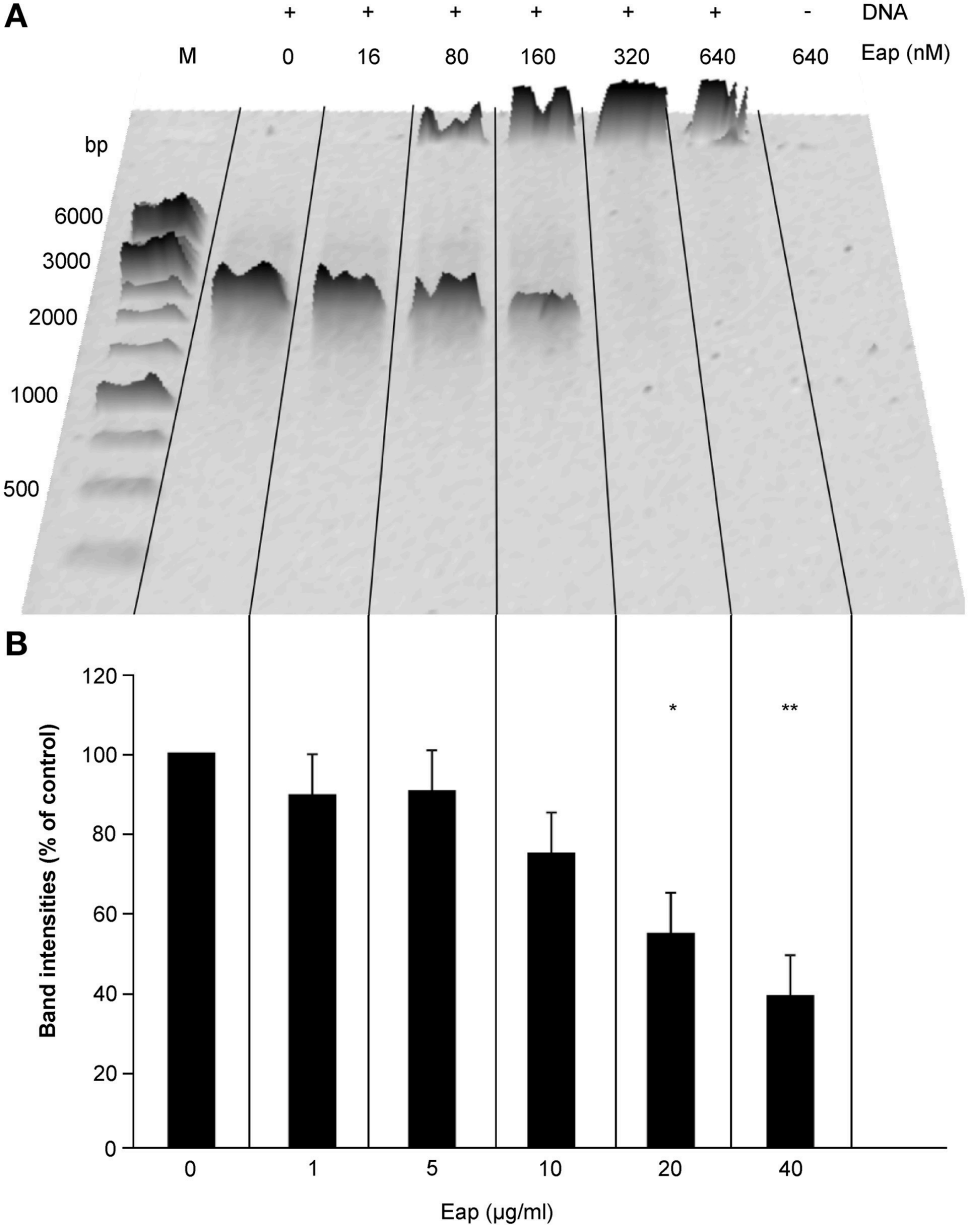
Interaction of Eap with DNA in a dose-dependent manner. Fifty Nanograms aliquots of a 1.4-kb PCR DNA-fragment were incubated for 5 min at 37°C with different concentrations of Eap as indicated and subjected to agarose gel electrophoresis. **(A)** Representative image of 0.8% agarose gel. M, DNA size marker. **(B)** Total band intensities of the DNA-dependent Sybr® Gold signals observed in wells and within the gel. Values are normalized to the signals seen with the Eap-free control sample, which was set to 100%. Data represent the mean ± SD of three independent experiments. *P < 0.05; **P < 0.01 (paired *t*-test between Eap-free control and Eap-treated groups).

The broad binding capacity of Eap and its cationic charge in solution (*pI* = 9.93 at pH 7.2) suggests that this bacterial exoprotein might also bind to (poly-) anionic molecules such as DNA. To test this hypothesis, we first incubated a defined amount of a 1.4-kbp DNA-PCR fragment (50 ng) with increasing concentrations of Eap (0, 1, 5, 10, 20, and 40 μg/ml or 0, 16, 80, 160, 320, and 640 nM, respectively), and subjected the mixtures to agarose gel electrophoresis (AGE). This analysis revealed a clear shift in the mobility of the DNA bands in the presence of Eap (Figure 1A), which completely prevented the migration of DNA into the gel at Eap concentrations ≥20 μg/ml. Thus, these findings indicate the formation of large DNA-protein aggregates, very likely due to binding of Eap to DNA. Moreover, densitometric evaluation of band intensities of separated DNA samples after AGE displayed a dose-dependent decrease in signal intensities in the presence of Eap (Figure 1B), indicating a partial loss of intact DNA during the separation on AGE. In controls, neither a shift in the mobility of DNA nor a clear drop in DNA amounts were seen when proteinase K-digested Eap (640 nM) or recombinant versions of the monomeric Eap homologs EapH1 and EapH2 (800 nM) were used (Figure S1).

### Eap binds to DNA but does not degrade DNA

**Figure 2:**
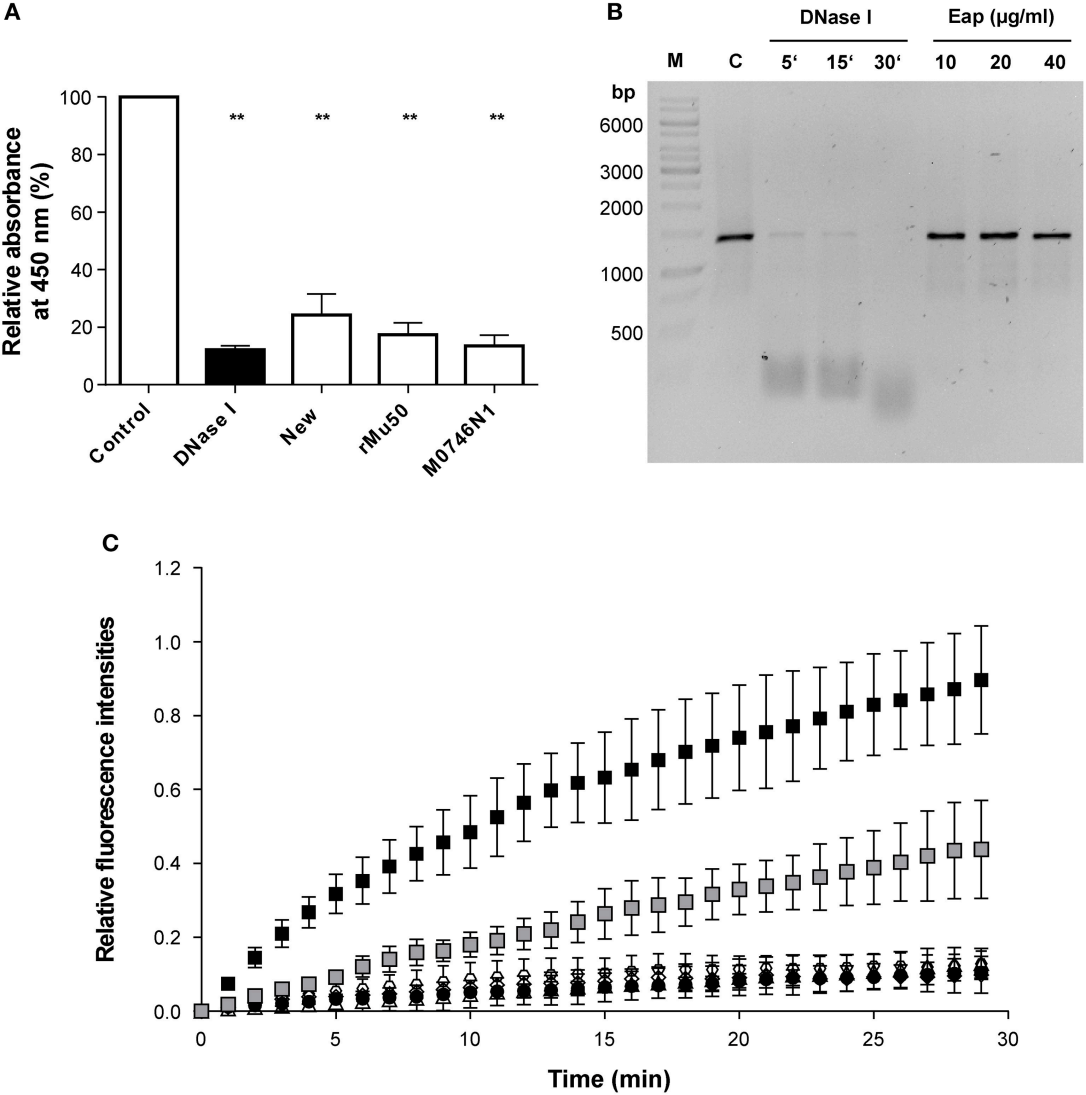
Influence of Eap on DNA stability. **(A)** DNase activities of different Eap (3 μg/ml) preparations. Eap samples were purified from strain Newman (New), its *nuc1 nuc2* derivative (M0746N1), or as a recombinant protein from *E. coli* (rMu50). DNase activity was quantified by ELISA (given in optical density (OD) readings at 450 nm). PCR grade water and DNase I (12.5 mKuU) served as negative and positive controls, respectively. OD 450 nm readings were normalized in relation to the values obtained for the negative controls, which were set to 100%. Data represent the mean ± SD of four to six independent experiments. **P < 0.01 (paired *t*-test between control and treated groups). **(B)** Fifty Nanograms of a 1.4-kbp DNA-PCR fragment was incubated with the Newman Eap preparation (10, 20, and 40 μg/ml) for 30 min at 37°C. Alternatively, 50 ng of the DNA-PCR fragment were co-incubated with 0.4 mKuU of DNase I for the time points indicated. Protein contents of the mixtures were subsequently removed by phenol/chloroform/isoamyl alcohol treatment, and DNA fractions subjected to AGE. A representative image of 0.8% agarose gel that was post stained with Sybr® Gold is shown. M, DNA size marker; C, untreated control. **(C)** Forty microgram per milliliter of Eap preparations from strain Newman (open triangles), its *nuc1 nuc2* derivative M0746N1 (open hexagons), or produced recombinantly from *E. coli* (open diamonds), and different amounts of a DNase I standard (0.2 and 2 mKuU; gray and black squares, respectively) were co-incubated with a FAM/BHQ-1 labeled oligonucleotide probe for up to 30 min at 37°C in a real time fluorescence reader. PCR grade water served as negative control (black circles). Fluorescence signals at 520 and 601 nm were recorded after every minute, and FAM-signals were normalized in relation to the ROX reference signals, and subsequently plotted in relation to the fluorescence signals seen at T0, which were set to 0. Data represent the mean ± SD of three independent experiments.

The partial decrease of the DNA signal after AGE with increasing concentrations of Eap indicated that this secreted bacterial protein might also exhibit some intrinsic DNase activity. To test this hypothesis, the influence of Eap on DNA was assayed with different DNase activity assays (Figure 2). At first, the DNase activities of different Eap (3 μg/ml) preparations, purified from strain Newman (New), its *nuc1 nuc2* derivative M0746N1, or as a recombinant protein from *E. coli* (rMu50), were quantified using a commercial immunometric enzyme immunoassay (Figure 2A). Similar to the positive control (12.5 mKuU of DNase I), clear decreases in absorbance rates at 450 nm were observed for all three Eap preparations, indicating that Eap might indeed possess some DNase activity or that this bacterial protein blocks the adhesion of the HRP-conjugated anti DNase substrate antibody to its target. Usage of the Eap preparation of the *nuc1 nuc2* derivative M0746N1 ruled out that this putative DNase activity may originate from a carry-over of *S. aureus* nucleases Nuc1 and Nuc2 in the Eap preparation obtained from strain Newman.

However, when 50 ng of the 1.4-kbp DNA-PCR fragment utilized for the EMSA were incubated with the Newman Eap preparation (10, 20, and 40 μg/ml) for 30 min at 37°C, and protein contents of the mixtures were subsequently removed by phenol/chloroform/isoamyl alcohol treatment prior to AGE, neither a decrease in DNA size nor a significant reduction in DNA amount was noticed (Figure 2B). In contrast, 0.4 mKuU of DNase I almost completely degraded the DNA probe in this period of time.

Next, the Eap preparations were tested with a commercial fluorescence based DNase activity assay (Figure 2C). Here, no relevant increase in fluorescence at 520 nm was observed with any of the Eap preparations, when compared to the negative control (PCR grade water), indicating that Eap did not degrade the FAM/BHQ-1 labeled oligonucleotide probe. In contrast, incubation of the dual labeled DNase probe with DNase I yielded increased fluorescence signals at this wavelength in a dose- and time-dependent manner.

Taken together, these findings strongly suggest that Eap has no apparent DNase activity and that the decrease in DNA content observed in the EMSA might be due to a partial binding of Eap-DNA complexes to the walls of the reaction tubes. The supposed DNase activity of Eap suggested by the immunometric enzyme immunoassay probably originated from a blocking of the DNase substrate by Eap, which prevented the binding of the HRP-conjugated anti-DNase substrate antibodies to the DNase substrate.

### Eap binds to linearized DNA and mediates aggregation of DNA

**Figure 3:**
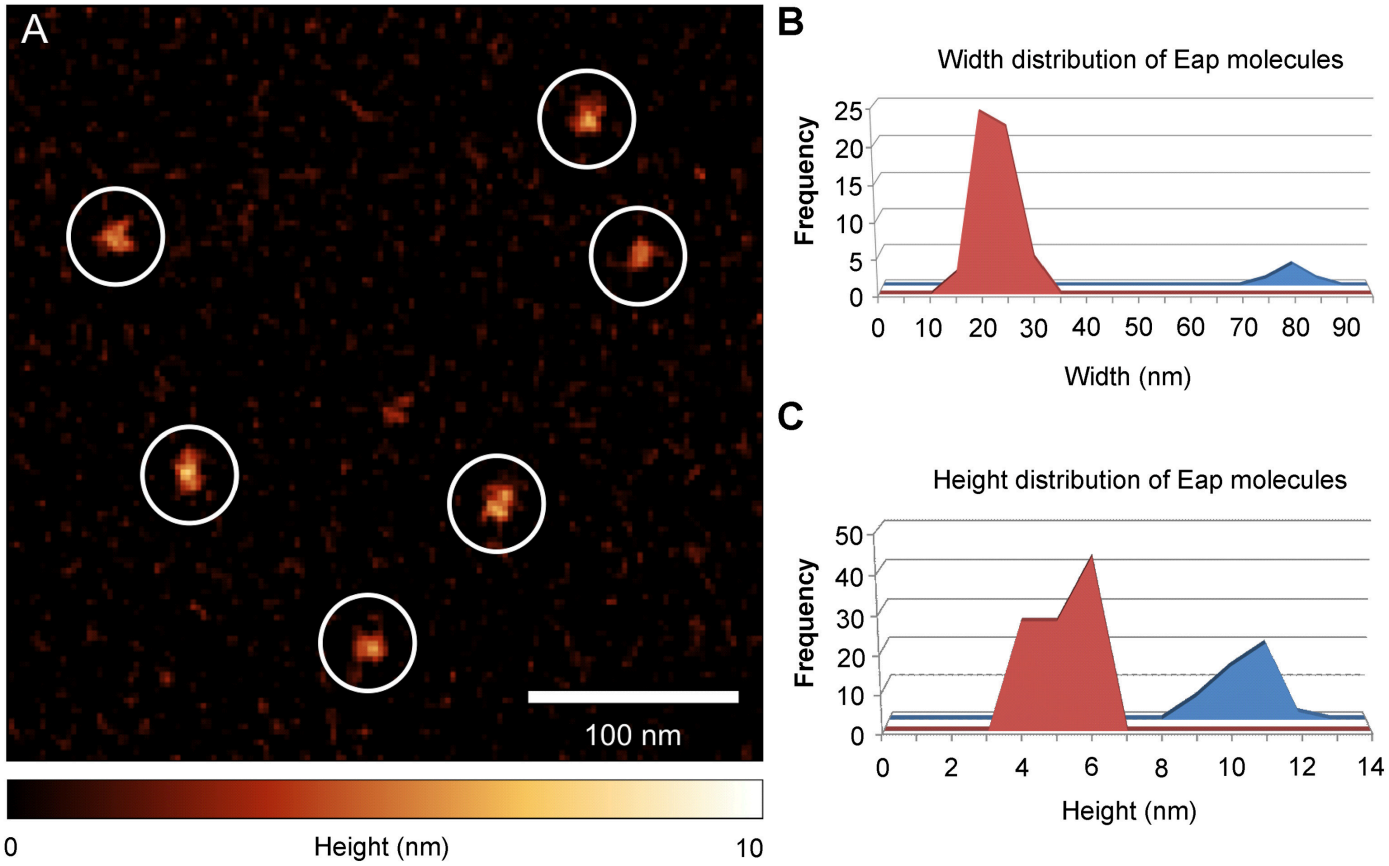
Eap shape distribution on OTS silicon membrane. Purified Eap (5 ng/ml) of strain Newman was spotted on octadecyltrichlorosilane (OTS) silicon and air dried prior to atomic force microscopy (AFM) imaging. **(A)** Representative AFM height image of Eap molecules (circles) adhering to the OTS modified surface. **(B,C)** Width and height distributions of OTS bound Eap molecules (*n* = 115).

Atomic force microscopy (AFM) was applied on DNA-Eap samples to foster the previous observations obtained with EMSA. Since its invention in 1985 (Binnig et al., 1986), AFM has repeatedly been shown in interaction analysis and size determinations to provide superior resolution capabilities on various length scales, from large tissue samples (Graham et al., 2010), to single cells and bacteria (Müller and Dufrêne, 2011; Loskill et al., 2014), down to the molecular level (Oesterhelt et al., 1999). Previously, DNA-protein complexes were already successfully recorded by AFM imaging on numerous occasions (Bezanilla et al., 1994; Abdelhady et al., 2003; Bennink et al., 2003; Hamon et al., 2007; Pastré et al., 2010; Shlyakhtenko et al., 2013; Lyubchenko et al., 2014). To this end, purified Eap was initially deposited on an octadecyltrichlorosilane (OTS) terminated silicon surface (Figure 3), followed by protein size evaluation. In the size distribution histograms two peaks with heights of 6 ± 1 nm and 11 ± 1 nm, respectively, and widths of the adhered proteins of about 20 nm and 80 nm were observed (Figures 3B,C). These results indicate that isolated Eap was present as a mixture of monomers and multimers with a molar ratio of 14:1. This is in line with a previous observation reporting Eap to be present predominantly as a monomer in solution that adopts an extended conformation (Hammel et al., 2007).

**Figure 4:**
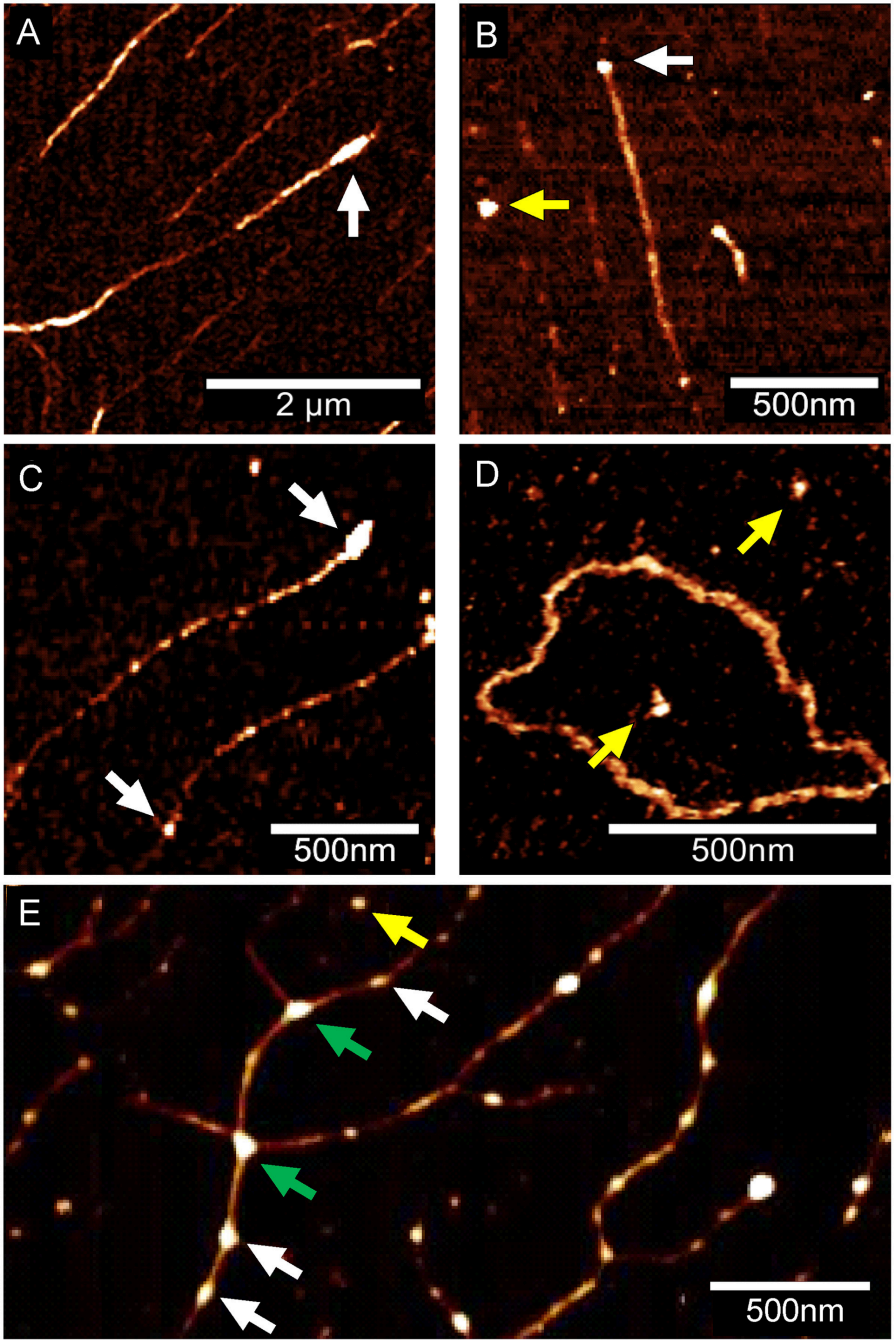
Eap binding to DNA is affected by the state and type of DNA. Eap (0.5 μg/ml) was co-incubated with DNA samples (100 ng/ml) obtained from different sources, and subsequently attached to OTS silicon prior to AFM imaging. Representative AFM height images of Eap/DNA complexes adhering to OTS silicon from three independent experiments are shown: **(A)** Phage lambda DNA. **(B)** Artificial 1.4 kb PCR DNA-product. **(C)** *Pst*I-digested plasmid pBR322 **(D)** Circular pBR322 isolated from *E. coli* DH5α. **(E)** Sheared herring sperm DNA. Eap molecules putatively adhering to DNA (white arrows), OTS (yellow arrows), or cross-linking DNA (green arrows) are indicated. DNA molecules displayed in **(A–C)**, and **(E)** were aligned to OTS silicon by DNA combing.

In the next step, different DNA/Eap mixtures were pre-incubated for 5 min at 37°C in PBS and subsequently spotted onto OTS terminated silicon surfaces, followed by analysis with AFM in the ScanAsyst® mode (Figure 4). Binding of Eap to one or both ends of linear DNA fragments (Phage Lambda DNA, PCR product, *Pst*I digested plasmid pPR322) was observed, whereas Eap bound rarely to circular DNA (pBR322) (Figures 4A–D, Figure S2), indicating a binding preference of Eap for open DNA ends. The nature of the DNA ends (blunt or sticky) appeared not to be of major importance, as pBR322 samples digested by *Pst*I (producing a sticky end with a 3′-overhang), by *Bam*HI (producing a sticky end with a 5′-overhang), or by *Eco*RV (producing a blunt end) were bound by Eap with comparable efficiencies (Figure S3). However, binding of sheared herring sperm DNA as a eukaryotic DNA source to Eap presents a different picture (Figure 4E). Indeed, Eap was frequently found to bind to this sheared herring sperm DNA on other positions than the fragments termini, potentially at DNA nicks caused by the shearing process. In addition, Eap tended to cross-link DNA-strands (green arrows, Figure 4E, Figure S2D) thereby forming a superstructure, in line with the previous EMSA-based findings, indicating that high concentrations of Eap promote the formation of large protein-DNA aggregates (see Figure 1A).

**Figure 5:**
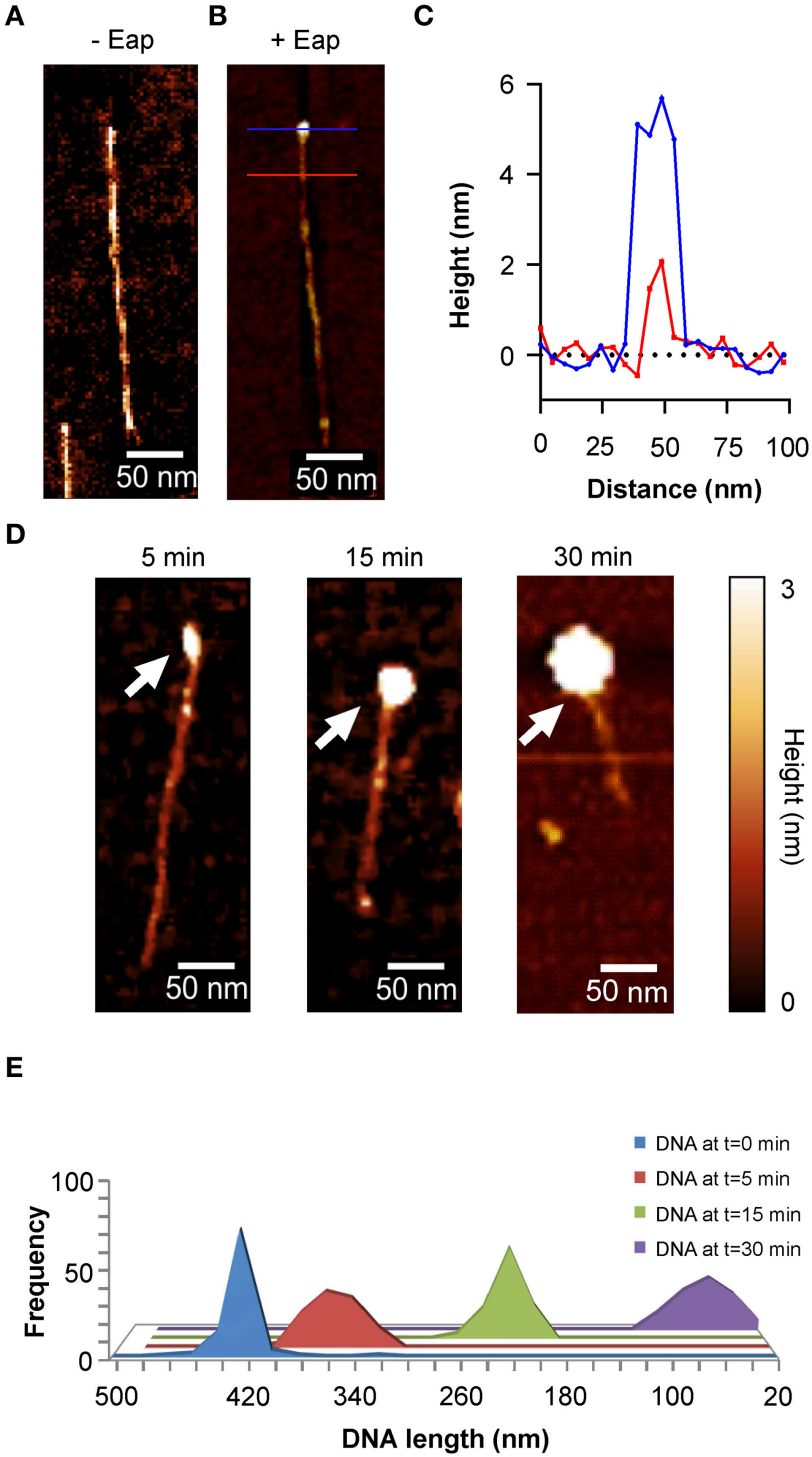
Aggregation of DNA by Eap. Eap (0.5 μg/ml) was co-incubated with 100 ng/ml of an 1.4-kb PCR DNA-fragment in PBS at 37°C and subsequently aligned to OTS silicon using DNA combing. **(A)** Representative AFM height image of an aligned PCR product without Eap. **(B)** AFM height image of a 1.4-kb PCR DNA-fragment bound by an Eap molecule. Blue line: Eap-DNA cross section; red line: DNA cross section. **(C)** Line profiles taken from the horizontal lines depicted in B. **(D)** Representative height images of Eap co-incubated with the 1.4-kb PCR DNA-fragment for the time points indicated. Arrows depict Eap-DNA-aggregates. **(E)** Histogram of the PCR DNA-fragment length decay in relation to the co-incubation time with Eap (*n* = 80–120 fragments per time point).

In order to determine the fate of such DNA-Eap complexes, DNA (100 ng/ml) was incubated with Eap (0.5 μg/ml) for different periods of time, and the DNA-protein samples were subsequently deposited onto the OTS terminated silicon surface using a combing technique (Allemand et al., 1997) that spreads the DNA fragments onto the surface in a highly ordered fashion (Figure 5A). DNA length measurements revealed a mean size of about 420 nm for the yielded PCR DNA product in the absence of Eap, which is in agreement with the reported values suggesting a length of 0.3 nm per base pair and a stretch rate of 105–120% (Bensimon et al., 1994). Moreover, cross sections of Eap-bound DNA revealed heights of 2 nm for the DNA fragment and 5–6 nm for DNA bound Eap molecules in most of the cases (Figure 5B and Figure S4). However, when the 1.4-kb PCR DNA fragment was incubated with Eap for increasing periods of time, a continuous decrease in DNA length over time was found (Figures 5C–E). Along with this, heights of the Eap-DNA complexes increased to 22 ± 5 nm after 30 min of co-incubation (Figure S4). These observations strongly indicate that Eap not only binds to DNA but also mediates its aggregation.

### Eap aggregates neutrophil-derived NETs

**Figure 6:**
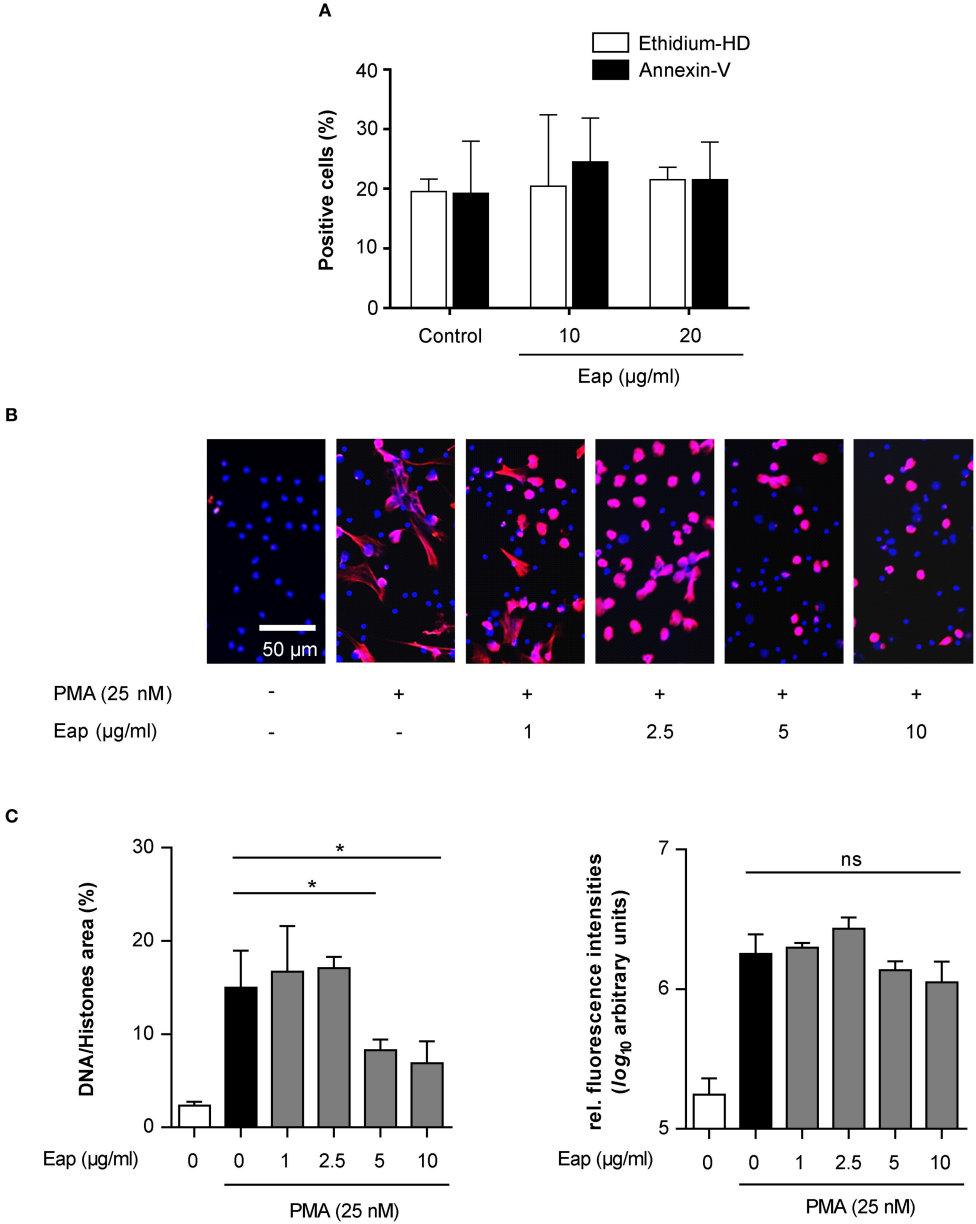
Eap interferes with NET formation in a dose-dependent manner. **(A)** Human neutrophils were incubated for 3 h with the Eap concentrations indicated, and apoptosis and necrosis were evaluated by determining the portion of cells positive for Annexin-V and Ethidium homodimer III, respectively. Data represent the mean ± SD of two independent experiments. **(B)** Human neutrophils were incubated for 3 h with PMA and different concentrations of Eap as indicated. Immunohistochemistry was performed to visualize NETs using an anti-DNA/histone H1 antibody (red), and DAPI (blue) for nuclear detection. The images are representative of three experiments with neutrophils from different donors. Please note that the DNA/histone antibody used for detection has a very high affinity for decondensated chromatin in NETs in comparison to DAPI (Saffarzadeh et al., 2014). **(C)** Quantification of the surface area covered by NETs and the anti-DNA/histone H1 antibody derived fluorescence signal intensities per 100 neutrophils. The data are presented as mean ± SD of three independent experiments done in duplicate. *p < 0.05; ns, not significant (paired *t*-test between PMA treated group and PMA + Eap challenged groups).

**Figure 7:**
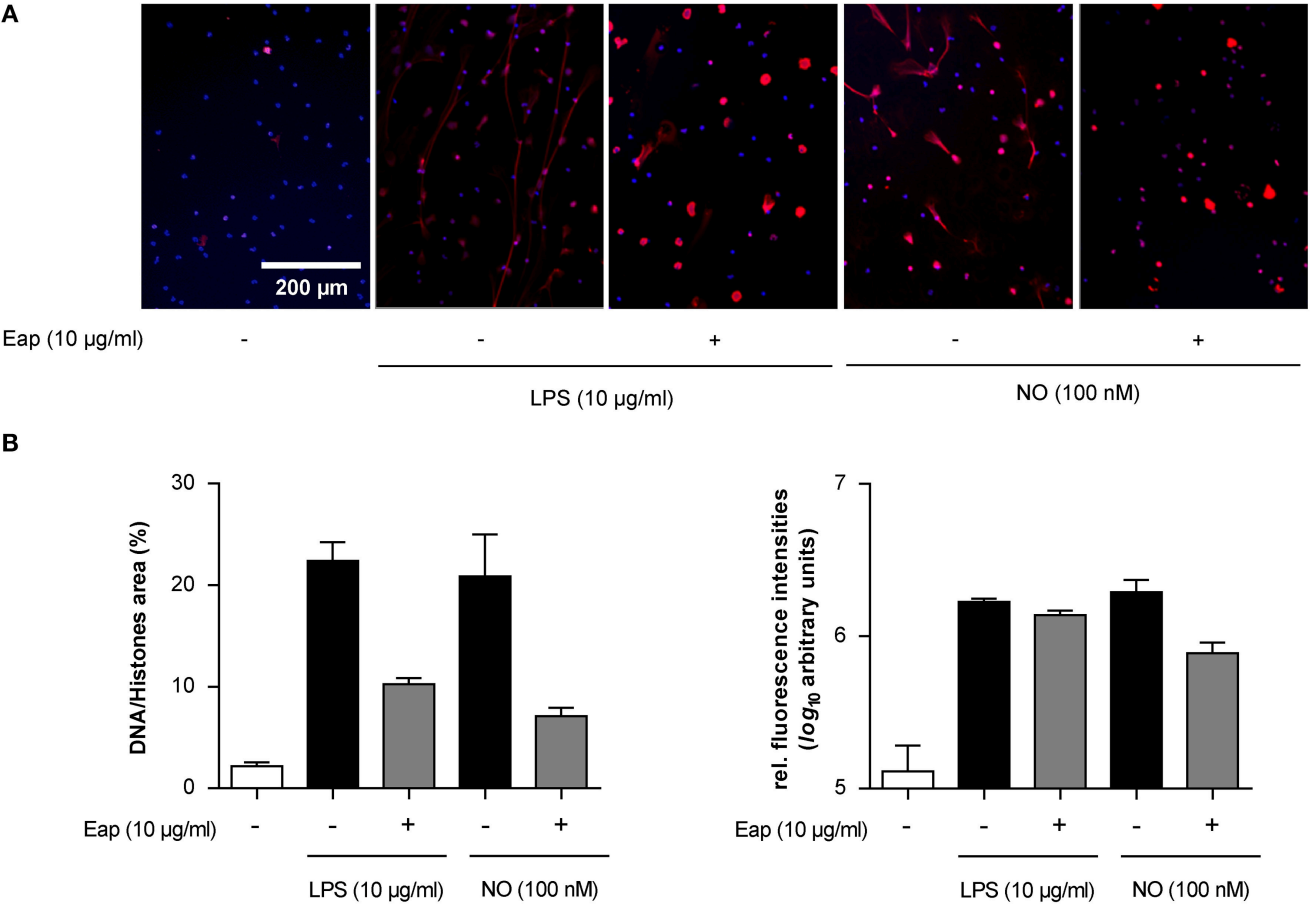
Eap modulates NET formation induced by different stimuli. Human neutrophils were incubated for 3 h with 10 μg/ml of LPS or 100 nM NO in absence (−) or presence (+) of 10 μg/ml Eap. Immunohistochemistry was performed to visualize NETs using an anti- DNA/histone H1 antibody (red), and DAPI (blue) for nuclear detection. **(A)** Representative fluorescence images of two independent experiments are shown. **(B)** Quantification of the surface area covered by NETs and the anti-DNA/histone H1 antibody derived fluorescence signal intensities per 100 neutrophils. The data are presented as mean ± SD of two independent experiments done in duplicate.

Next, in order to test the DNA aggregation capacity of Eap in another biological context, its ability to interact with neutrophil-derived NETs was evaluated (Figure 6). In the absence of agents that may induce neutrophil stimulation, Eap alone did not promote necrosis or apoptosis of neutrophils (Figure 6A). However, following induction of neutrophils by phorbol 12-myristate 13-acetate (PMA) to trigger the production of extracellular DNA/histone complexes, denoted “neutrophil extracellular traps” (NETs), co-incubation with isolated Eap resulted in a dose-dependent diminution of such networks, documented by the loss of the characteristic staining pattern of NETs (Figures 6B,C). Instead, increased staining rates were observed at the cell surfaces and/or the nuclei of stimulated neutrophils in the presence of Eap, suggesting the presence of larger aggregates of decondensed DNA in these regions (Figure 6B). Moreover, Eap also markedly reduced the formation of NETs when neutrophils were activated by other stimuli such as lipopolysaccharide (LPS) or a nitric oxide (NO) donor (Figure 7).

Together, these data indicate that Eap contains a DNA binding capacity that interferes with the formation of natural extracellular DNA structures such as NETs independent of the stimulus for neutrophil-related NETosis.

### Eap can alter preformed NETs

**Figure 8:**
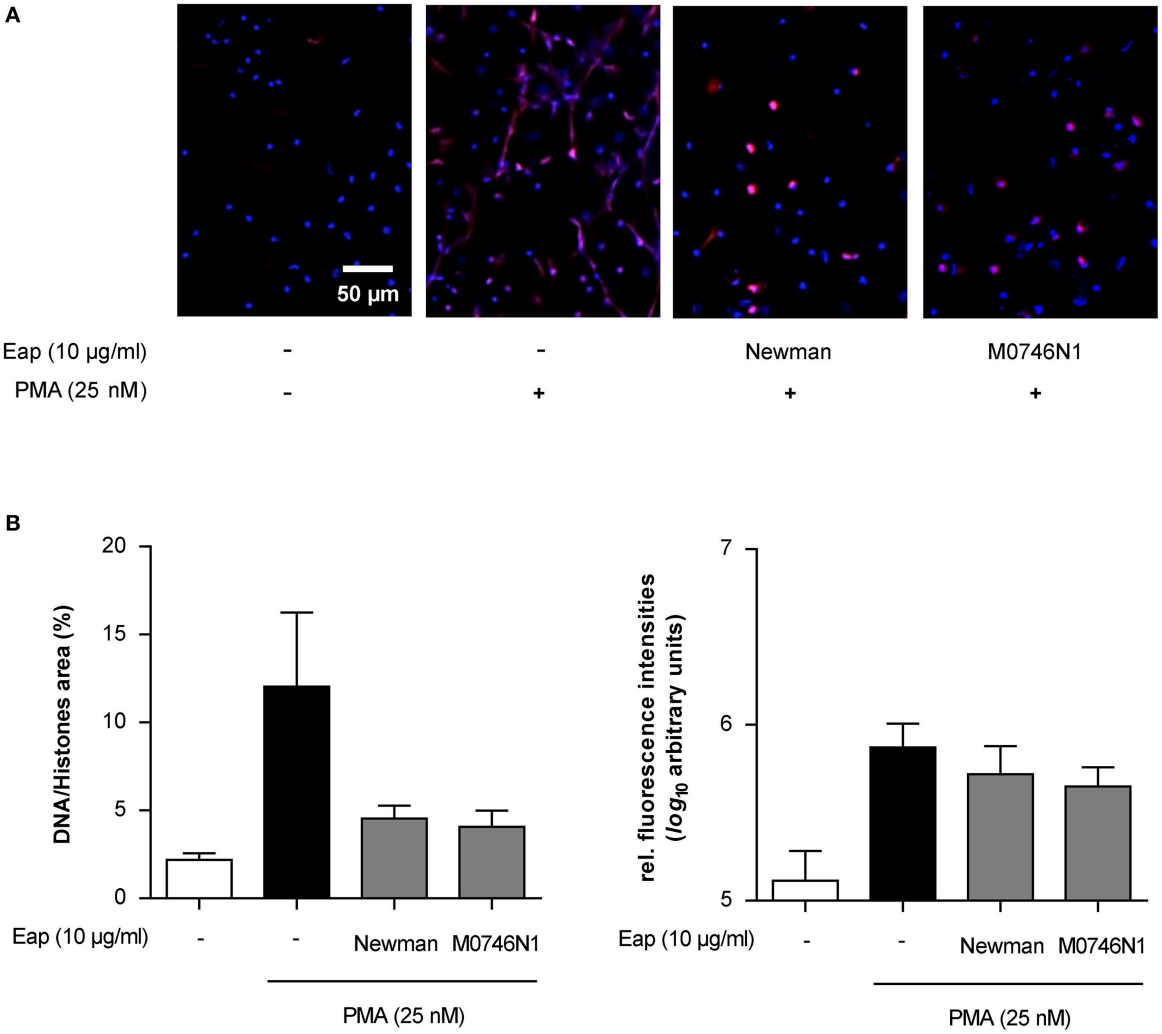
Eap effect on preformed NETs. Neutrophils were incubated for 2 h with PMA to produce NETs, and subsequently challenged for 1 h with Eap (10 μg/ml) from strain Newman and its *nuc1 nuc2* derivative (M0746N1), respectively. Immunohistochemistry was performed to visualize NETs using an anti-DNA/histone H1 antibody (red) and DAPI (blue) for nuclear detection. **(A)** Representative fluorescence images of two independent experiments are shown. **(B)** Quantification of the surface area covered by NETs and the anti-DNA/histone H1 antibody derived fluorescence signal intensities per 100 neutrophils. The data are presented as mean ± SD of two independent experiments done in duplicate.

*S. aureus* is known to secrete at least two heat stable endonucleases, termed Nuc1 and Nuc2 (Tang et al., 2008) that, like Eap, are expressed in a SaeRS-dependent manner and are primarily produced during the later stages of bacterial growth (Harraghy et al., 2005; Olson et al., 2013). To confirm the specificity of Eap's intrinsic DNA aggregation activity and to exclude that the decreased number of NETs observed in the presence of isolated Eap was caused by a contamination with such nucleases, we also challenged NETs with purified Eap from the *S. aureus* Newman derivative M0746N1 (Kaito et al., 2011), lacking both nucleases. Both Eap samples were tested in parallel experiments, whereby each Eap sample was added to the neutrophils 2 h post induction of NETosis by PMA (Figure 8). Importantly, both Eap preparations were able to decrease the area covered by preformed NETs (Figure 8B). Together, these findings strongly indicate that Eap not only interferes with NET formation but can also modulate the stability of preformed NETs, and that this NET interfering capacity of Eap was not due to contamination with nucleases.

## Conclusions

The secreted adhesive protein Eap of *S. aureus* is a multifunctional protein that exerts a number of immunomodulatory functions (Chavakis et al., 2005), particularly with regard to protection of bacteria against host defense machineries. These include inhibition of leukocyte recruitment to the site of infection, anti-angiogenic function to block revascularization of wounds or protease inhibition (Chavakis et al., 2007). Here, we present a novel activity of isolated Eap, documented by its ability to bind to DNA and to modulate the formation and stability of NETs.

In particular, our data indicates that Eap preferentially binds to the termini of linearized DNA and expresses an intrinsic DNA aggregation activity. This DNA binding activity of Eap appears to be compatible with Eap's functions to protect *S. aureus* against NET-mediated extracellular trapping (Menegazzi et al., 2012; Azzouz et al., 2018). Together with its recently described activity as neutrophil serine protease inhibitor in association with NET functions (Stapels et al., 2014), Eap possesses another property as a DNA aggregation factor to prevent extracellular trapping by this important host defense mechanism. Finally, NETs are considered to be a primary component of arterial and venous thrombi and thereby help to capture circulating bacteria to prevent their dissemination into tissue (Massberg et al., 2010). Conversely, due to its NET-aggregation capacity, Eap may thereby protect bacteria from being trapped in microthrombi, allowing them to continue their infectious process in the body.

## Ethics statement

All healthy individuals gave a written informed consent for blood donation, approved by the Ethics Committee of the Medical Faculty of the Justus-Liebig-University, Giessen, Germany (file numbers 05/00 and 178/2011BO1).

## Author contributions

JE, MS, HP, PJ, and NT: performed experiments, analyzed data, and contributed to the preparation of figures. MB and KP: conceived and coordinated the study. JE, MS, HP, KJ, MH, VM, KP, and MB: designed research. JE, MS, HP, KP, BG, and MB: wrote the paper. All authors edited the manuscript.

### Conflict of interest statement

The authors declare that the research was conducted in the absence of any commercial or financial relationships that could be construed as a potential conflict of interest.
