# Supplementary material for: The Staphylococcus aureus Extracellular Adherence Protein Eap Is a DNA Binding Protein Capable of Blocking Neutrophil Extracellular Trap Formation
Source: Front Cell Infect Microbiol. 2018 Jul 9;8:235. doi: 10.3389/fcimb.2018.00235 (PMC6047304; doi:10.3389/fcimb.2018.00235)
Supplement: Supplementary file 1 [file Image_1.PDF]

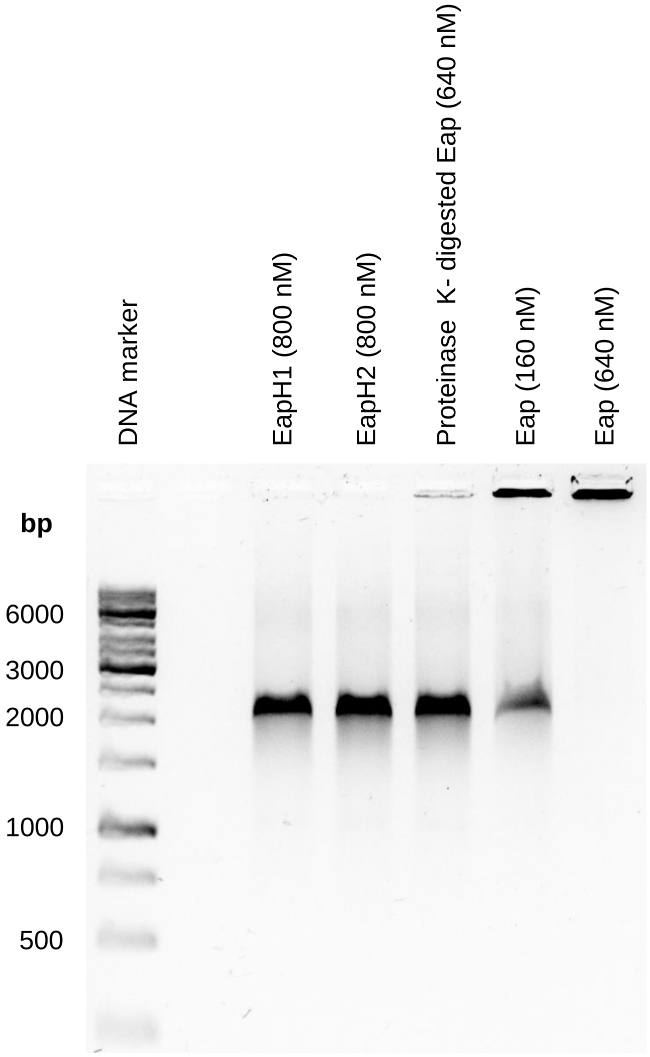

Figure S1: Eap homologs EapH1 and EapH2 do not shift DNA. 50 ng aliquots of a 1.4-kb PCR DNA-fragment were incubated for 5 min at 37°C with different preparations of Eap as indicated, and subsequently subjected to agarose gel electrophoresis. A representative image of an 0.8% agarose gel is shown.

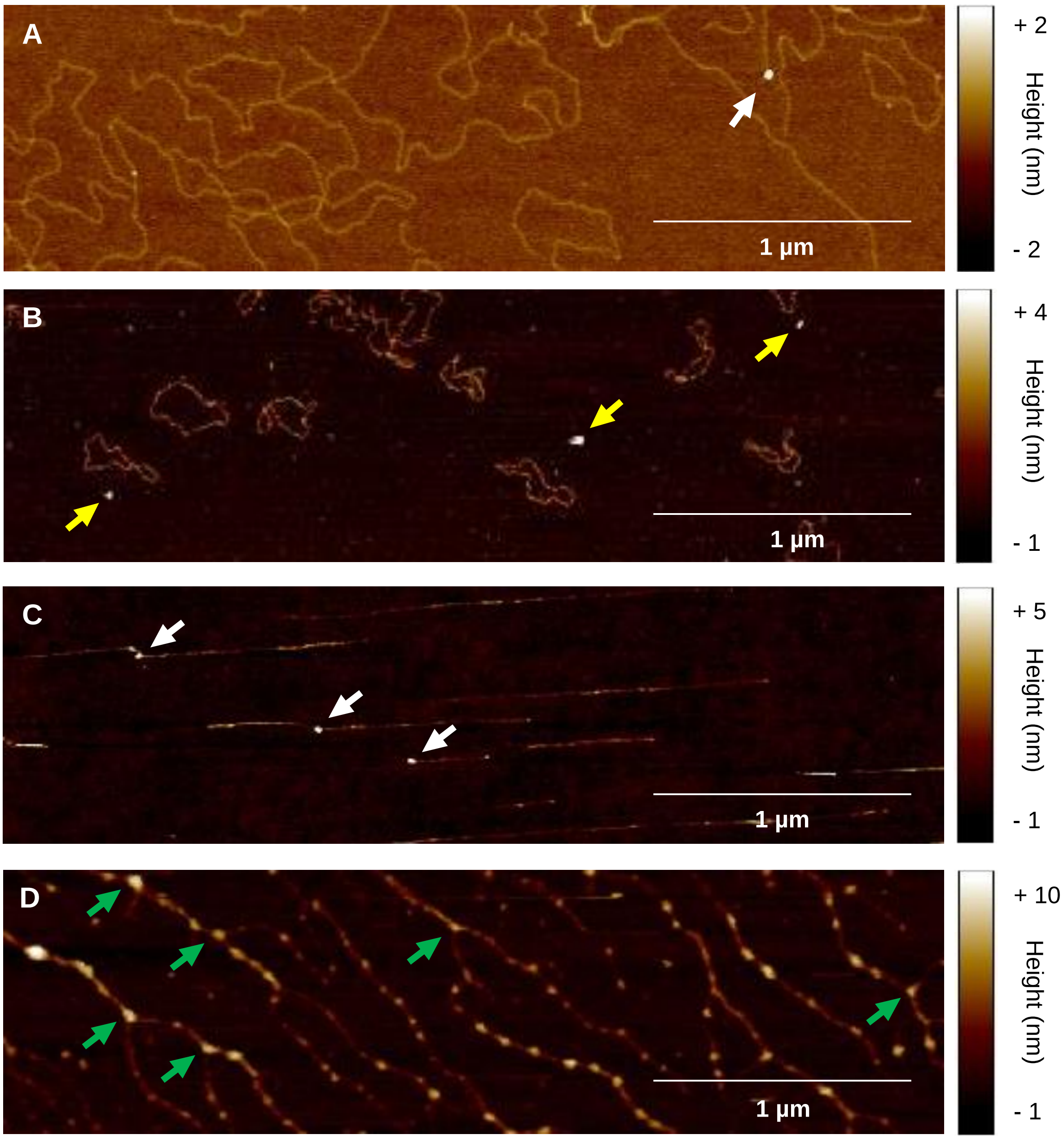

Figure S2: AFM height images of DNA molecules co-incubated with Eap. DNA samples (100 ng/ml) obtained from different sources were co-incubated with Eap (0.5 µg/ml) for 5 min, and subsequently attached to OTS silicon prior to AFM imaging. Representative height images of AFM scans (1 x 4 µm) of Eap and/or DNA adhering to OTS silicon from three independent experiments are shown: **(A)** Phage lambda DNA. **(B)** Circular plasmid pBR322. **(C)** *Pst*I-digested plasmid pBR322. (**D**) Sheared herring sperm DNA. Selected Eap molecules putatively adhering to DNA (white arrows), OTS (yellow arrows), or cross-linking DNA (green arrows) are indicated. DNA molecules displayed in C and D were aligned to OTS silicon by DNA combing.

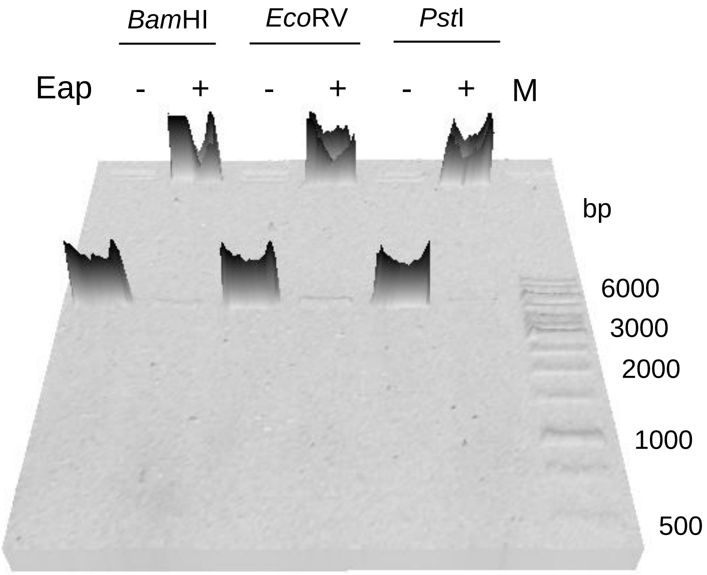

Figure S3: Blunt- and sticky-ended DNA molecules are bound by Eap with comparable efficiencies. 50 ng aliquots of pBR322 digested with the restriction enzymes *Bam*HI (producing a sticky end with a 5'-overhang), *Eco*RV (producing a blunt end), and *Pst*I (producing a sticky end with a 3'-overhang), respectively, were incubated for 5 min at 37°C in absence (-) or presence (+) of Eap (160 nM), and subsequently subjected to agarose gel electrophoresis. A representative image of an 0.8% agarose gel is shown. M, marker.

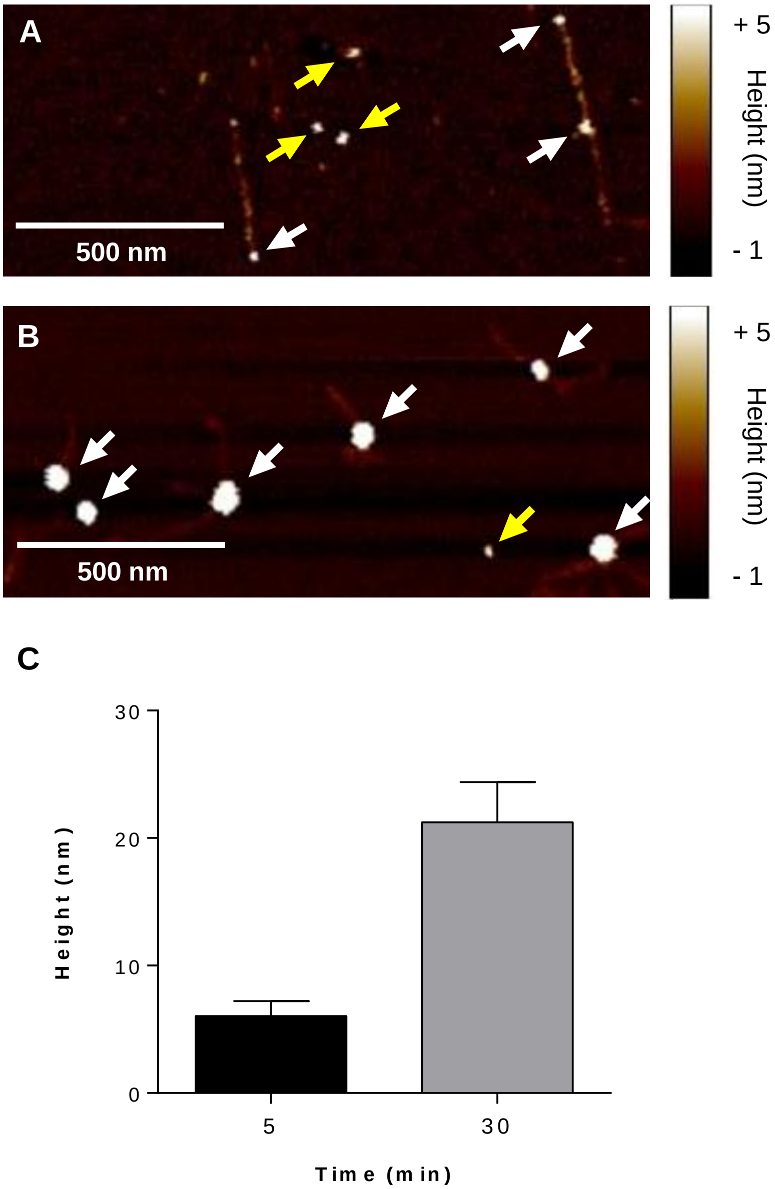

Figure S4: Aggregation of DNA by Eap. 100 ng/ml of an 1.4-kb PCR DNA-fragment was co-incubated with Eap (0.5 µg/ml) in PBS at 37°C and subsequently aligned to OTS silicon using DNA combing. **(A, B)** Representative AFM height images of aligned PCR products co-incubated for 5 min (A) and 30 min (B) with Eap, respectively. White arrows depict putative Eap molecules bound to DNA-fragments, yellow arrows DNA-free Eap. **(C)** Heights of the Eap-DNA complexes in relation to the co-incubation time with Eap (*n*=25 Eap-DNA complexes per time point).
